# Supplementary figures and images for: Genetic drift promotes and recombination hinders speciation on holey fitness landscapes
Source: PLoS Genet. 2024 Jan 22;20(1):e1011126. doi: 10.1371/journal.pgen.1011126 (PMC10833538; doi:10.1371/journal.pgen.1011126)

**A**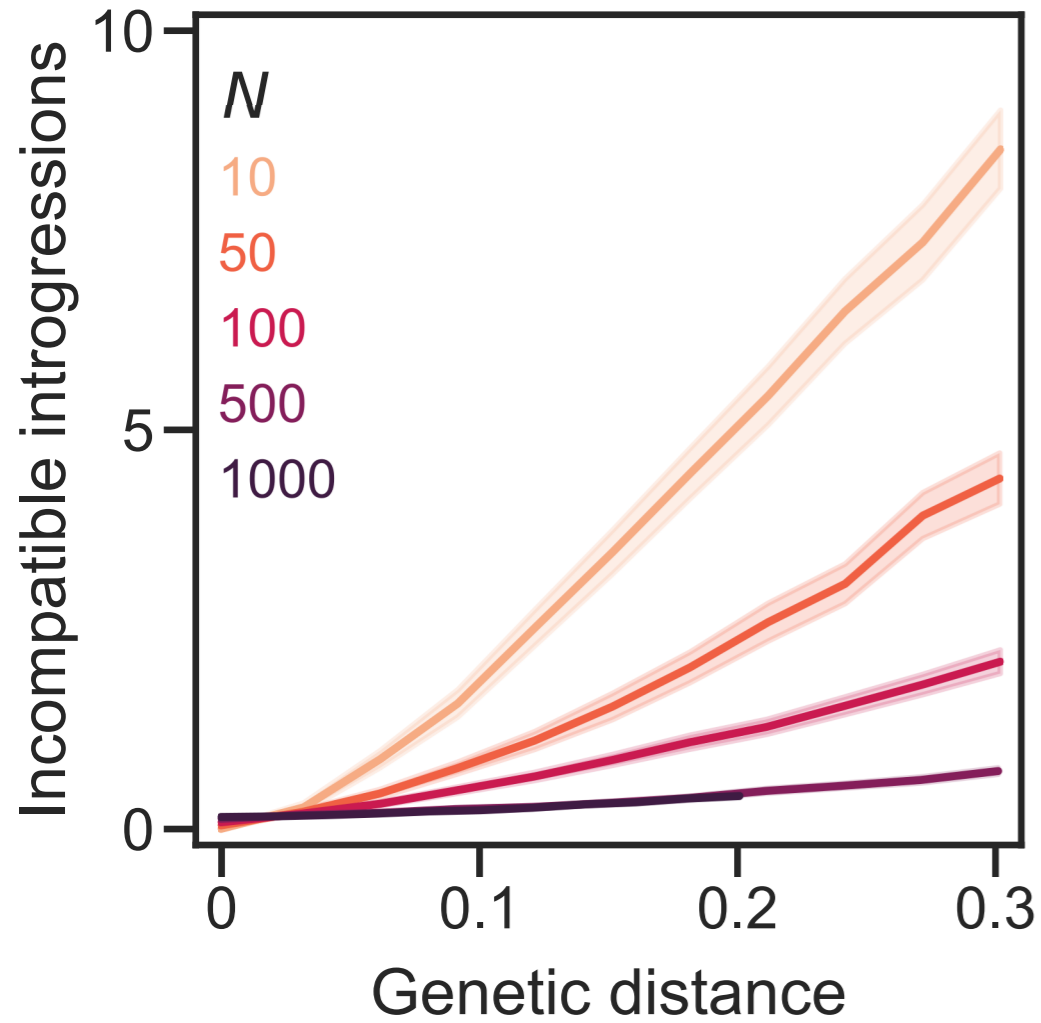**B**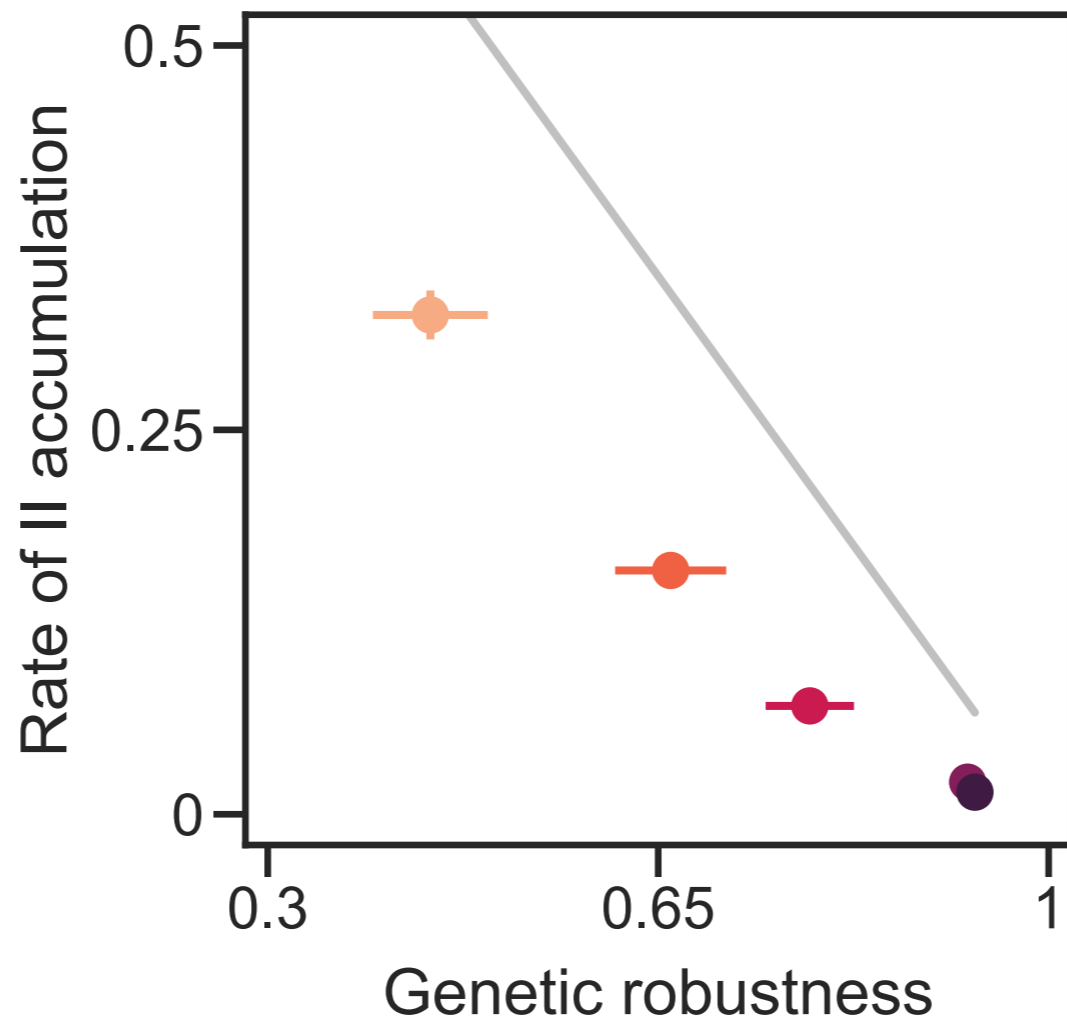**C**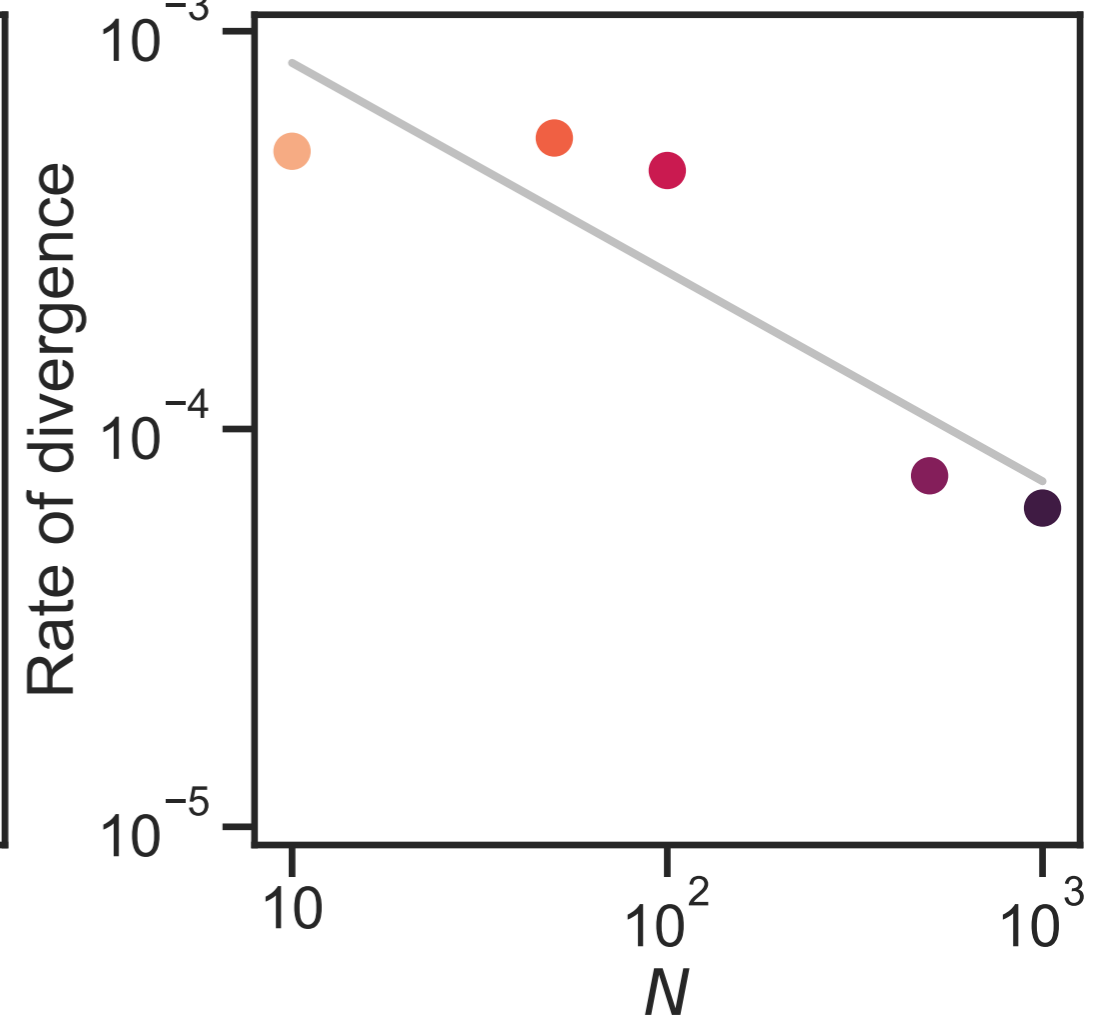

Supplement: S1 Fig — (A) IIs accumulated approximately linearly with D in sexual populations of different sizes (N). In all simulations, populations evolved under the RNA folding model (with σ = 0 and L = 100) and experienced U = 0.1, random mating, and free recombination. (B) IIs accumulated faster in smaller populations because they evolved lower ν. The gray line shows b/L = 1 − ν (see Eq 1). (C) Large populations diverged more slowly. The gray line shows a power law with exponent −0.5. Plotted values in all panels are means of 200 replicate simulation runs at each N. Error bands in (A) and bars in (B) are 95% CIs (in (C) they are hidden by the points). See Fig 7 for more details. (PDF) [file pgen.1011126.s001.pdf]

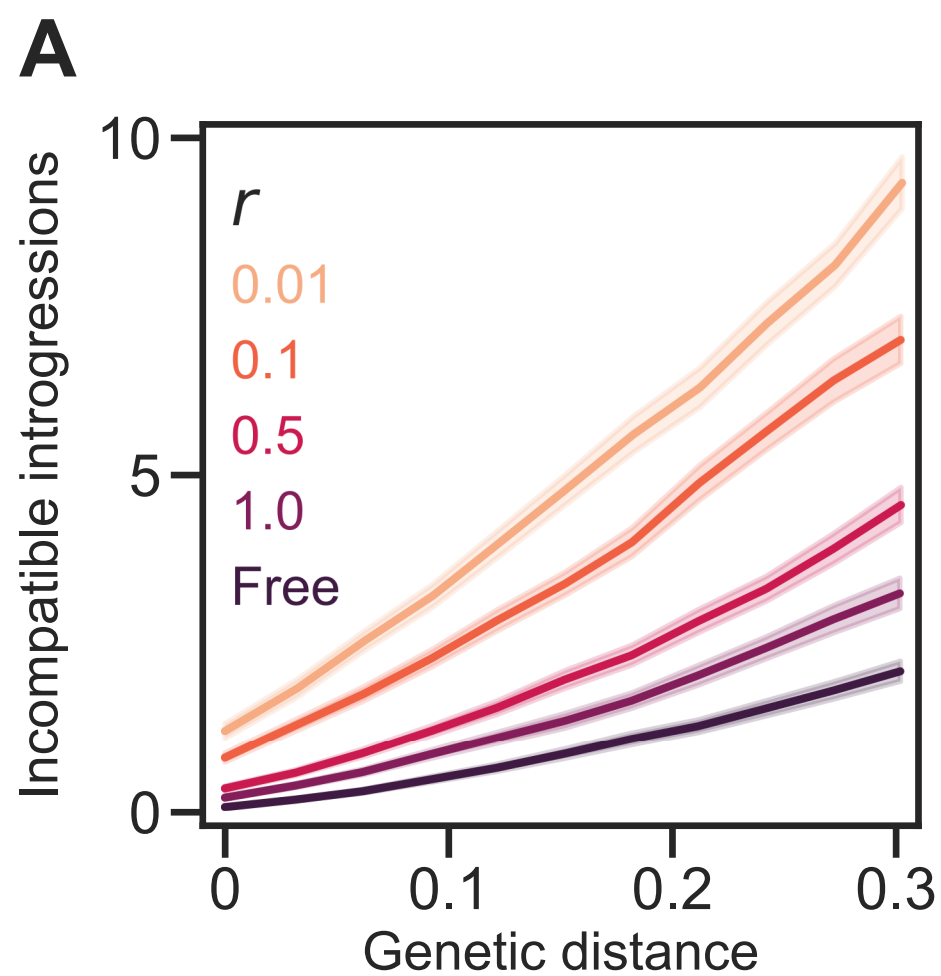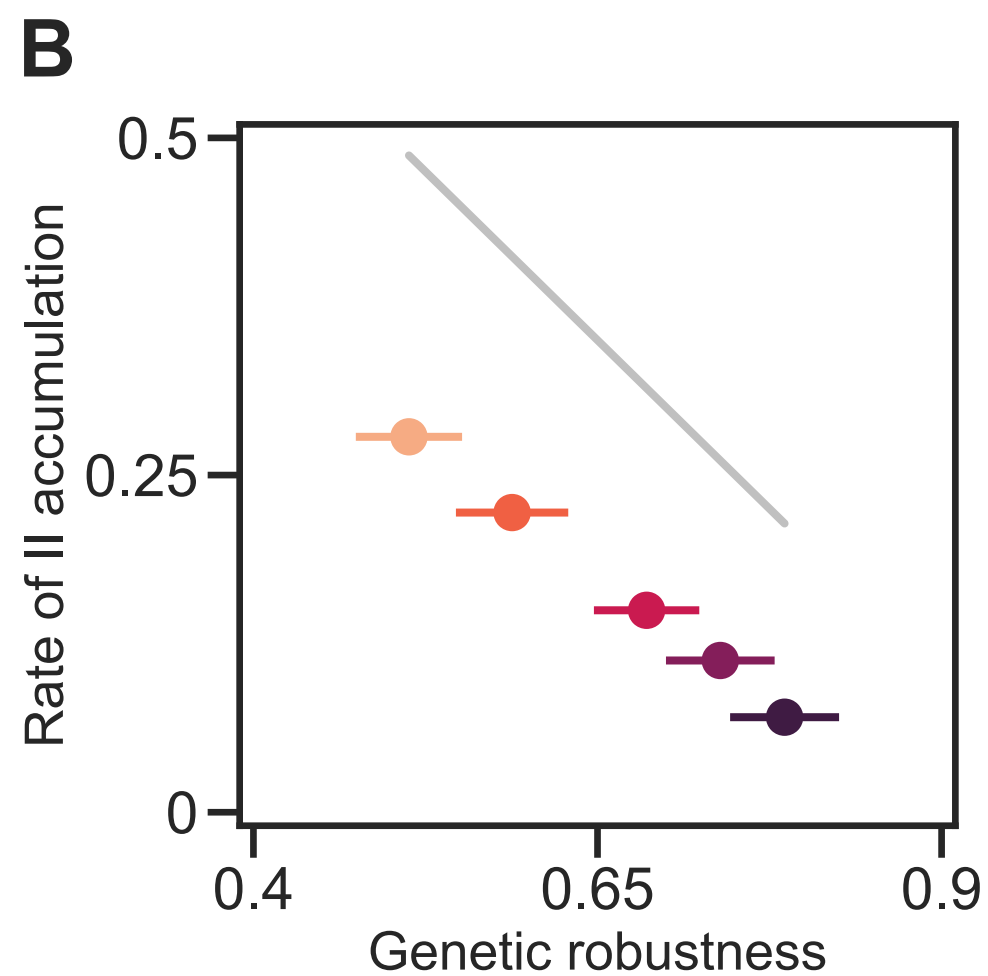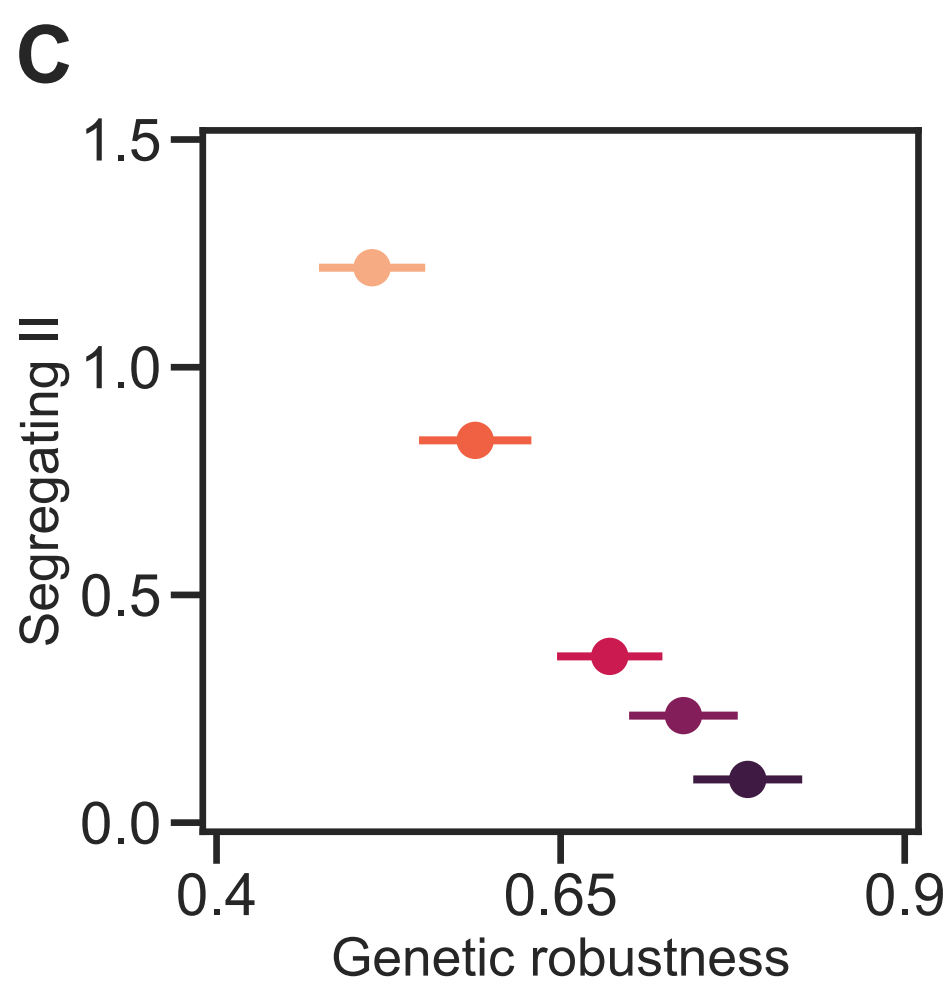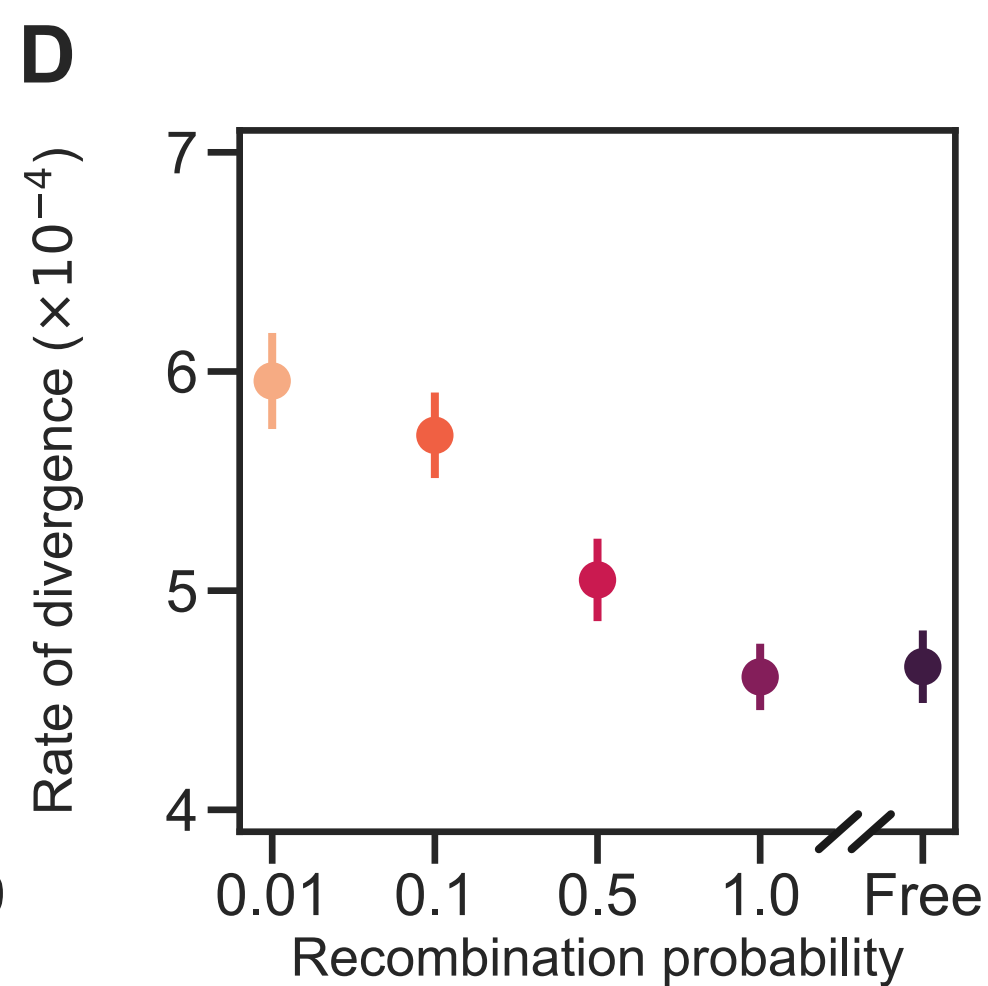

Supplement: S2 Fig — (A) IIs accumulated approximately linearly with D in populations of N = 100 individuals experiencing different recombination probabilities. In all simulations, populations evolved under the RNA folding model (with σ = 0 and L = 100) and experienced U = 0.1 and random mating. (B) IIs accumulated faster in populations experiencing low recombination probability because they evolved lower ν. The gray line shows b/L = 1 − ν (see Eq 1). (C) Populations experiencing low recombination probability accumulated more segregating IIs. (D) Populations experiencing high r diverged more slowly. Values show the rate of increase in D per generation. Plotted values in all panels are means of 200 replicate simulation runs at each r. Error bands in (A) and bars in (B)–(D) are 95% CIs (some are hidden by the points). See Fig 8 for more details. (PDF) [file pgen.1011126.s002.pdf]

**A**

Recipient

Donor

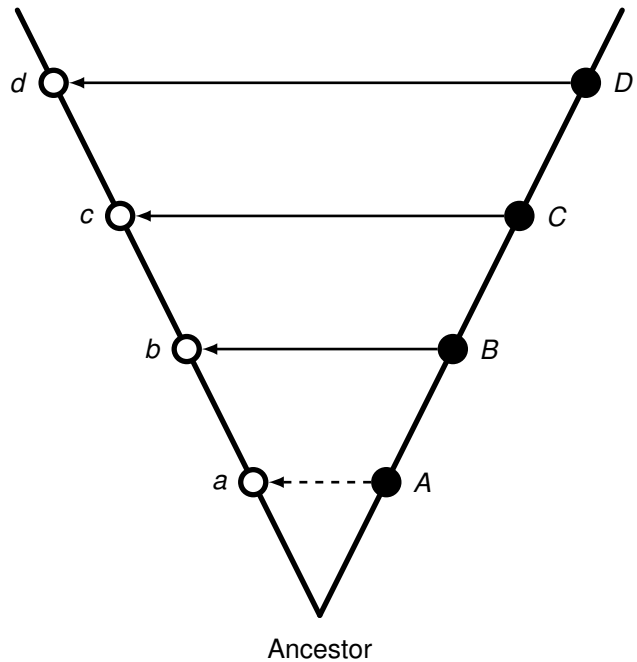**B**

Recipient

Donor

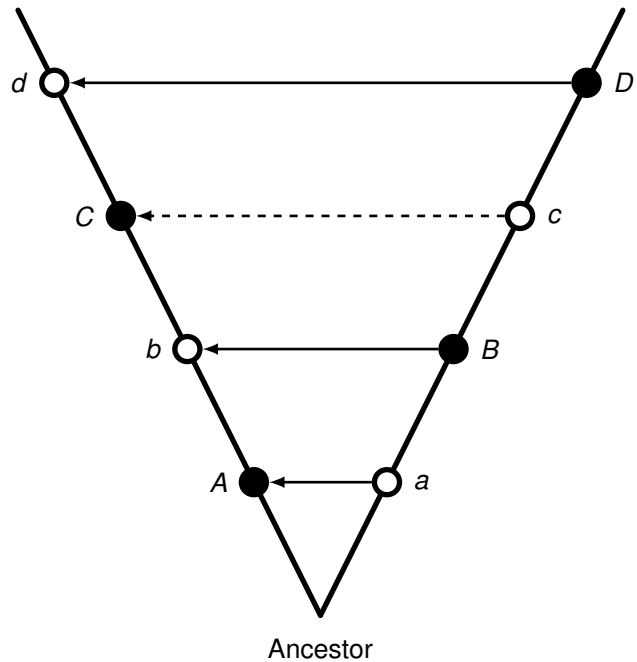

Supplement: S3 Fig — (A) Example of scenario A. Two populations diverge in allopatry. Both populations are initially fixed for lowercase alleles (open circles) at four loci (abcd). Derived alleles are indicated by uppercase letters (closed circles). The donor population undergoes four substitutions, fixing the ABCD genotype. The recipient population does not undergo any substitutions, retaining the ancestral genotype abcd. Arrows indicate introgressions of divergent alleles from the donor population to the recipient population. Three introgressed genotypes have not been tested by natural selection (aBcd, abCd, and abcD; solid arrows) but one has (Abcd, dashed arrow). (B) Example of scenario B. Each population undergoes two substitutions, the recipient population fixing the AbCd genotype and the donor population fixing the aBcD genotype. Three introgressed genotypes have not been tested by natural selection (abCd, ABCd, and AbCD; solid arrows) but one has (Abcd, dashed arrow). (PDF) [file pgen.1011126.s003.pdf]
