# Supplementary material for: Genetic drift promotes and recombination hinders speciation on holey fitness landscapes
Source: PLoS Genet. 2024 Jan 22;20(1):e1011126. doi: 10.1371/journal.pgen.1011126 (PMC10833538; doi:10.1371/journal.pgen.1011126)
Supplement: S1 Text — (PDF) [file pgen.1011126.s004.pdf]

S1 Text. Number of divergent alleles from one population that have not have been tested by natural selection in the genetic background of another population

If two populations evolving in allopatry have diverged at k diallelic loci and there have been no reversals, then $k - 1$ divergent alleles from one population will not have been tested by natural selection in the genetic background of the other population. The number of alleles is $k - 1$ rather than k because we must exclude one allele that *has* been tested in the recipient background. This point explains both the $k - 1$ and $\tilde{\nu}$ terms in Eq 1. Which allele is to be excluded depends on the number of substitutions that have taken place in the recipient population. There are two possible scenarios, A and B (S3 Fig).

Scenario A: If no substitutions have taken place in the recipient population we exclude the introgression of the derived allele at the first locus to undergo a substitution in the donor population because the resulting genotype has been tested by natural selection (S3A Fig, dashed arrow).

Scenario B: If at least one substitution has taken place in the recipient population we exclude the introgression of the ancestral allele at the last locus to undergo a substitution in the recipient population because the resulting genotype has been tested by natural selection (S3B Fig, dashed arrow; also, Fig 1B).
