# Supplementary material for: Genetic drift promotes and recombination hinders speciation on holey fitness landscapes
Source: PLoS Genet. 2024 Jan 22;20(1):e1011126. doi: 10.1371/journal.pgen.1011126 (PMC10833538; doi:10.1371/journal.pgen.1011126)
Supplement: S1 Table — (PDF) [file pgen.1011126.s005.pdf]

S1 Table. Synonymous nucleotide diversity (πS) at the *Adh* locus for different species of *Drosophila* .

group	species	πS	sequences	reference	notes
ananassae	*ananassae*	0.015822	10	1	
obscura	*bogotana*	0.004841	8	2	
obscura	*miranda*	0.003703	12	3	
obscura	*persimilis*	0.013317	6	4	
obscura	*pseudoobscura*	0.016660	139	2, 5, 6	
obscura	*subobscura*	0.023465	16	7	
virilis	*americana*	0.034155	19	8	G96X
virilis	*americana*	0.023301	19	8	G96Y
virilis	*borealis*	0.059733	2	9, 10	
virilis	*lummei*	0.000000	2	9, 11	
virilis	*texana*	0.029706	10	8	
virilis	*virilis*	0.005490	2	11	
repleta	*arizonae*	0.013155	13	12	Adh-1
repleta	*arizonae*	0.052620	11	12	Adh-2
repleta	*buzzatii*	0.016323	4	13	
repleta	*mojavensis*	0.013141	13	12	Adh-1
repleta	*mojavensis*	0.012597	15	14	Adh-1
repleta	*mojavensis*	0.021064	13	12	Adh-2
repleta	*mojavensis*	0.009859	15	14	Adh-2
repleta	*hydei*	0.017564	9	13	
repleta	*mulleri*	0.019467	6	13	
immigrans	*albomicans*	0.031127	16	15	
immigrans	*hypocausta*	0.031060	2	16	
immigrans	*nasuta*	0.003687	16	15	
immigrans	*siamana*	0.038144	2	16, 17	
melanogaster	*lutescens*	0.009242	4	18–21	
melanogaster	*mauritiana*	0.002228	6	22	
melanogaster	*melanogaster*	0.024036	10	23	
melanogaster	*melanogaster*	0.027122	4	24	
melanogaster	*melanogaster*	0.030007	11	25	
melanogaster	*sechellia*	0.000000	2	22	
melanogaster	*simulans*	0.027407	7	26	
melanogaster	*yakuba*	0.007072	12	26	
melanogaster	*yakuba*	0.015629	36	27	
willistoni	*equinoxialis*	0.005490	2	28	
willistoni	*nebulosa*	0.011506	4	28, 29	
willistoni	*paulistorum*	0.018475	3	28	
willistoni	*willistoni*	0.018035	18	30, 31	
montium	*auraria*	0.045728	2	32	

montium	*birchii*	0.051745	2	32
montium	*kikkawai*	0.034314	21	33
montium	*leontia*	0.027858	2	32
montium	*lini*	0.062418	2	32
montium	*rufa*	0.000000	2	32
montium	*serrata*	0.022432	2	32
montium	*triauraria*	0.011194	2	32
montium	*triauraria*	0.019847	3	34

Methods: Sequences were obtained from GenBank. Synonymous nucleotide diversity (πS) was calculated using the Nei-Gojobori method in MEGA-CC version 11.0.13 (35).

References: 1) Shih & Jones 2008 Genetics 180: 1261–1263. 2) Shaeffer & Miller 1991 PNAS 88: 6097–6101. 3) Yi et al. 2003 Genetics 164: 1369–1381. 4) Wang et al. 1997 Genetics 147: 1091–1106. 5) Schaeffer & Miller 1993 Genetics 135: 541–552. 6) Schaeffer 2002 Genet. Res. 80: 163–175. 7) Jones et al. 2005 Genetics 170: 207–219. 8) McAllister & Charlesworth 1999 Genetics 153: 221–233. 9) Nurminsky et al. 1996 Mol. Biol. Evol. 13: 132–149. 10) Morales-Hojas 2011 Mol. Phyl. Evol. 60: 249–258. 11) Wang et al. 2006 Mol. Phyl. Evol. 40: 484–500. 12) Matzkin & Eanes 2003 Genetics 163: 181–194. 13) Begun 1997 Genetics 145: 375–382. 14) Matzkin 2004 Mol. Biol. Evol. 21: 276–285. 15) Satomura & Tamura 2016 Mol. Biol. Evol. 33: 367–374. 16) Katoh et al. 2007 Zool. Sci. 24: 913–921. 17) Rice et al. 2018 Evol. Dev. 20: 78–88. 18) Katoh et al. 2000 J. Mol. Evol. 51: 122–130. 19) O'Grady & Kidwell 2002 Mol. Phyl. Evol. 22: 442–453. 20) Ko, David & Akashi 2003 J. Mol. Evol. 57: 562–573. 21) Katoh & Watada 2015 GenBank: LC057203.1. 22) Kliman et al. 2000 Genetics 156: 1913–1931. 23) Begun et al. 1999 Mol. Biol. Evol. 16: 1816–1819. 24) Laurie et al. 1991 Genetics 129: 489–499. 25) Kreitman 1983 Nature 304: 412–417. 26) McDonald & Kreitman 1991 Nature 351: 652–654. 27) Siddiq et al. 2017 Nat. Ecol. Evol. 1: 0025. 28) Gleason et al. 1998 Evolution 52: 1093–1103. 29) Gao et al. 2011 Mol. Phyl. Evol. 60: 98–107. 30) Anderson et al. 1993 Mol. Biol. Evol. 10: 605–618. 31) Griffith & Powell 1997 J. Mol. Evol. 45: 232–237. 32) Chen et al. 2013 Zool. Sci. 30: 1056–1062. 33) Goto et al. 2004 Genes Genet. Syst. 19–26. 34) Dai, Lu, Lv, Chen, Cheng & Zhang 2001 GenBank: AF348879.1, AF348880.1, AF348882.1. 35) Kumar et al. 2012 Bioinformatics 28: 2685–2686.
