# Supplementary material for: Genetic drift promotes and recombination hinders speciation on holey fitness landscapes
Source: PLoS Genet. 2024 Jan 22;20(1):e1011126. doi: 10.1371/journal.pgen.1011126 (PMC10833538; doi:10.1371/journal.pgen.1011126)
Supplement: S2 Table — (PDF) [file pgen.1011126.s006.pdf]

S2 Table. Total recombination map length for different species of *Drosophila* .

group	species	map length (cM)	reference	mapping function	corrected†
ananassae	*ananassae*	350.1	1	Kosambi	yes
immigrans-tripunctata	*funebris*	895.6	1	Kosambi	yes
immigrans-tripunctata	*mediopunctata*	609.6	1	Kosambi	yes
melanogaster	*mauritiana*	488.5	2	Foss	yes
melanogaster	*melanogaster*	294.9	1	Kosambi	yes
melanogaster	*melanogaster*	287.3	3	N/A*	no
melanogaster	*simulans*	426.2	4	Kosambi	yes
montium	*serrata*	282.3	5	Kosambi	yes
obscura	*persimilis*	605.1	1	Kosambi	yes
obscura	*pseudoobscura*	557.1	1	Kosambi	yes
obscura	*subobscura*	1007.6	1	Kosambi	yes
repleta	*buzzatii*	696.5	1	Kosambi	yes
repleta	*hydei*	655.2	1	Kosambi	yes
virilis	*montana*	632.7	6	Kosambi	yes
virilis	*virilis*	732.3	7	N/A*	no
willistoni	*willistoni*	320.4	8	Kosambi	yes

* High resolution mapping.

† The length of each chromosome was multiplied by $(n + 1) / (n - 1)$, where n is the number of markers per chromosome.

References: 1) Cáceres et al. 1999 Genetics 153: 251–259. 2) True et al. 1996 Genetics 142: 507–523. 3) Comeron et al. 2012 PLoS Genetics 8: e1002905. 4) Barker & Moth 2001 Dros. Inf. Serv. 84: 205–206. 5) Stocker et al. 2012 G3 2: 287–297. 6) Schäfer et al. 2010 J. Evol. Biol. 23: 518–527. 7) Hemmer et al. 2020 Mobile DNA 11: 10. 8) Spassky & Dobzhansky 1950 Heredity 4: 201–215.
