# Supplementary material for: Genetic drift promotes and recombination hinders speciation on holey fitness landscapes
Source: PLoS Genet. 2024 Jan 22;20(1):e1011126. doi: 10.1371/journal.pgen.1011126 (PMC10833538; doi:10.1371/journal.pgen.1011126)
Supplement: S3 Table — (PDF) [file pgen.1011126.s007.pdf]

S3 Table. Phylogenetic signal for each trait analyzed in Fig. 10.

trait	Blomberg's K	P-value	Pagel's λ	$P(\lambda=0)$	$P(\lambda=1)$
nucleotide diversity	0.430	0.928	0.000	1.000	0.018
map length	1.124	0.056	1.155	0.156	0.175
postzygotic RI velocity	0.427	0.957	0.000	1.000	0.017

Methods: The phylogeny shown in Fig. 10A was used for all analyses.
Blomberg's K and corresponding P-value were calculated using the R package `phytools` version 1.0-3 (1). The P-value was calculated from 100,000 randomizations.
Pagel's λ was estimated by maximum likelihood using `phytools`. P values were calculated by likelihood ratio tests. The log-likelihood for $\lambda=0$ was obtained from `phytools`; the log-likelihood for $\lambda=1$ was calculated using the R package `geiger` version 2.0.10 (2).

References: 1) Revell 2012 Meth. Ecol. Evol. 3: 217–223. 2) Pennell et al. 2014 Bioinformatics 30: 2216–2218.
