# Supplementary material for: Genetic drift promotes and recombination hinders speciation on holey fitness landscapes
Source: PLoS Genet. 2024 Jan 22;20(1):e1011126. doi: 10.1371/journal.pgen.1011126 (PMC10833538; doi:10.1371/journal.pgen.1011126)
Supplement: S4 Table — (PDF) [file pgen.1011126.s008.pdf]

S4 Table. Asymmetry in synonymous nucleotide diversity (πS) at the *Adh* locus and in postzygotic reproductive isolation (RI) for different species of *Drosophila* .

species 1	species 2	group	πS1	πS2	πS asym	RI asym	RI12*	RI21*	references	notes
arizonae	*mojavensis*	*repleta*	0.032887	0.014165	0.018722	0.334	0.500	0.166		mean of 4 estimates
auraria	*triauraria*	*montium*	0.045728	0.015520	0.030208	1.000	1.000	0.000		
auraria	*subauraria*	*montium*	0.045728	0.011091	0.034637	0.500	1.000	0.500	1	
equinoxialis	*paulistorum*	*willistoni*	0.005489	0.018475	-0.012986	-0.500	0.500	1.000		
lini	*ogumai*	*montium*	0.062418	0.022183	0.040235	0.000	0.500	0.500	1	
lini	*ohnishii*	*montium*	0.062418	0.027833	0.034585	0.000	0.500	0.500	1	
mauritiana	*sechellia*	*melanogaster*	0.002228	0.000000	0.002228	0.000	0.500	0.500		
ogumai	*ohnishii*	*montium*	0.022183	0.027833	-0.005650	0.000	0.500	0.500	1	
paulistorum	*willistoni*	*willistoni*	0.018475	0.018035	0.000440	0.250	1.000	0.750		
persimilis	*pseudoobscura*	*obscura*	0.013317	0.016660	-0.003343	0.000	0.500	0.500		
bogota	*pseudoobscura*	*obscura*	0.004841	0.016660	-0.011819	0.500	0.500	0.000		
recens	*subquinaria*	*quinaria*	0.063736	0.066094	-0.002358	-0.450	0.500	0.950	2	*Adhr* not *Adh*
simulans	*sechellia*	*melanogaster*	0.027407	0.000000	0.027407	0.000	0.500	0.500		
simulans	*mauritiana*	*melanogaster*	0.027407	0.002228	0.025179	0.000	0.500	0.500		
triauraria	*quadraria*	*montium*	0.015520	0.023228	-0.007708	-0.500	0.000	0.500	3, 4	

* 12 = species 1 ♀ ⨯ species 2 ♂; 21 = species 2 ♀ ⨯ species 1 ♂. Data from Table S2 of Yukilevich 2012 Evolution 66: 1430–1446.

References for estimates of πS: 1) Chen et al. 2013 Zool. Sci. 30: 1056–1062. 2) Ginsberg et al. 2019 J. Evo. Biol. 32: 1093–1105.
3) Dai, Lu, Lv, Chen, Cheng & Zhang 2001 GenBank: AF348883.1. 4) Watada & Miyake 2011 GenBank: AB669833.1.
All other estimates of πS are summarized in S1 Table.
