# Supplementary material for: Genetic drift promotes and recombination hinders speciation on holey fitness landscapes
Source: PLoS Genet. 2024 Jan 22;20(1):e1011126. doi: 10.1371/journal.pgen.1011126 (PMC10833538; doi:10.1371/journal.pgen.1011126)
Supplement: Attachment — Submitted filename: response.pdf [file pgen.1011126.s009.pdf]

December 11, 2023

Dear Drs Lindi Wahl and Gregory Barsh,

We are grateful for the opportunity to revise our manuscript and for the thoughtful comments from you and the reviewers. We have done our best to address every comment and feel the manuscript is much stronger as a result. We also thank you for giving us additional time to complete our revision.

Below, we respond to each of the comments individually; our responses are shown immediately below each comment. We have submitted a version of the manuscript showing substantive changes in red as a supplementary document.

We hope that you will find that the manuscript has improved through our efforts to address the comments provided. Please do not hesitate to contact us if we can clarify any of our responses further.

Yours sincerely,

Ricardo Azevedo
On behalf of the other authors

Response to the Editors' and Reviewers' Comments

Below, we reproduce the editors' and reviewers' comments in their entirety and address them one-by-one. We numbered them continuously in order of appearance. We subdivided and renumbered some individual comments for clarity.

Editors (Drs Lindi Wahl and Gregory Barsh)

E.1 Thank you very much for submitting your Research Article entitled 'Genetic drift promotes and recombination hinders speciation on a holey fitness landscape' to PLOS Genetics.

The manuscript was fully evaluated at the editorial level and by independent peer reviewers. Based on the reviews, we will not be able to accept this version of the manuscript, but we would be willing to review a much-revised version. We cannot, of course, promise publication at that time.

As you will see below, all three reviewers provided detailed, thoughtful feedback and found much to like about this manuscript. They also each identified substantial concerns. Among these, it will be critical to put the work in better context of work that has come before (including a clarification of "holey landscape" vs "neutral network"), and to test the robustness of your results to a number of assumptions, as suggested in the reviews. Key points to be addressed from the reviews include:

- disentangling the effects of drift vs. mutational supply by simulating at fixed NU
- addressing whether the results are apparent because the only selection that can occur is for robustness (if there were intrinsic fitness differences, would these swamp selection for robustness?)
- going further to address how robust the results are to variation in parameter values and the fitness landscape
- recomputing data for Figs 3 and 4, and addressing concerns re: the snowball exponent as suggested by Reviewer 2

Response: We believe we have addressed each of the "substantial concerns" and "key points" outlined above:

(a) "Put the work in better context of work that has come before (including a clarification of "holey landscape" vs "neutral network")."

See responses to R1.1, R1.2, and R2.7.

(b) "Disentangling the effects of drift vs. mutational supply by simulating at fixed NU."

See response to R1.4.

(c) "Test the robustness of [our] results to a number of assumptions. [...] Addressing whether the results are apparent because the only selection that can

occur is for robustness (if there were intrinsic fitness differences, would these swamp selection for robustness?). Going further to address how robust the results are to variation in parameter values and the fitness landscape."

See responses to R1.4, R1.5(a), R1.5(d), R1.10, and R3.2.

(d) "[R]ecomputing data for Figs 3 and 4, and addressing concerns re: the snowball exponent as suggested by Reviewer 2."

See responses to R2.2 and R2.5.

E.2 Should you decide to revise the manuscript for further consideration here, your revisions should also address the specific points made by each reviewer. We will also require a detailed list of your responses to the review comments and a description of the changes you have made in the manuscript.

Response: Below, we reproduce the reviewers' comments in their entirety and address them one-by-one. We numbered them continuously in order of appearance. We subdivided and renumbered some individual comments for clarity.

E.3 If you decide to revise the manuscript for further consideration at PLOS Genetics, please aim to resubmit within the next 60 days, unless it will take extra time to address the concerns of the reviewers, in which case we would appreciate an expected resubmission date by email to plosgenetics@plos.org.

If present, accompanying reviewer attachments are included with this email; please notify the journal office if any appear to be missing. They will also be available for download from the link below. You can use this link to log into the system when you are ready to submit a revised version, having first consulted our Submission Checklist.

To enhance the reproducibility of your results, we recommend that you deposit your laboratory protocols in protocols.io, where a protocol can be assigned its own identifier (DOI) such that it can be cited independently in the future. Additionally, PLOS ONE offers an option to publish peer-reviewed clinical study protocols. Read more information on sharing protocols at https://plos.org/protocols?utm_medium=editorial-email&utm_source=authorletters&utm_campaign=protocols

Please be aware that our data availability policy requires that all numerical data underlying graphs or summary statistics are included with the submission, and you will need to provide this upon resubmission if not already present. In addition, we do not permit the inclusion of phrases such as "data not shown" or "unpublished results" in manuscripts. All points should be backed up by data provided with the submission.

While revising your submission, please upload your figure files to the Preflight Analysis and Conversion Engine (PACE) digital diagnostic tool. PACE helps ensure that figures meet PLOS requirements. To use PACE, you must first register as a user. Then, login and navigate to the UPLOAD tab, where you will find detailed instructions on how to use the tool. If you encounter any issues or have any questions when using PACE, please email us at figures@plos.org.

PLOS has incorporated Similarity Check, powered by iThenticate, into its journal-wide submission system in order to screen submitted content for originality before publication. Each PLOS journal undertakes screening on a proportion of submitted articles. You will be contacted if needed following the screening process.

To resubmit, use the link below and 'Revise Submission' in the 'Submissions Needing Revision' folder.

We are sorry that we cannot be more positive about your manuscript at this stage. Please do not hesitate to contact us if you have any concerns or questions.

> **Response:** Noted.

Reviewer #1

R1.1 This paper uses simulations of computationally folded RNA populations to evaluate how epistatic incompatibilities accumulate between allopatric populations under the same selection pressures. This is a really interesting topic, and to my knowledge the inclusion of mutational robustness as an evolving factor in this study is relatively novel and leads to insightful results. While I have some notes on the framing, my major concerns are with the approach and interpretation of results. I don't think these concerns are necessarily indicative of serious problems, but give me pause about recommending the paper as written.

The Introduction is brief and skips past most literature connecting micro- and macroevolution. I don't think it is necessary to get into a big review of the literature, but as written the Introduction first seems to suggest that few have tried to link micro and macro, then focuses entirely on the accumulation of DMIs, which is, of course, one of the ways people have tried to link them.

> **Response:** We have attempted to make it clear that there has been extensive research into the mechanistic connection between micro- and macroevolution (lines 8–13). We cite two comprehensive reviews of the field [1,2] and highlight a specific example.

R1.2 Prior work on holey fitness landscapes and speciation is mentioned only very briefly in the Intro, and I think here you really should give a little more space to what has been done and what still needs to be done in this arena.

> **Response:** We now devote most of the Introduction to the topics of holey fitness landscapes and neutral networks (starting line 16).

R1.3 On the actual approach, I guess my first question is why we care about DMIs in asexual populations at all? Maybe I'm out of the loop here, but I don't see much relevance of the DMI concept to asexual species, which, as we see in Figure 3a, accumulate DMIs even without allopatry. I understand that the point is maybe to compare to sexual populations, but that seems adequately done by reducing r down toward zero, as is done in Figure 4. As such, the whole way the Results begin is a little baffling to me, and could I think be made more concise.

Response: We removed most work on asexual populations from the paper. Now, asexual populations are only used in the simulations summarized in Fig 9B and described in lines 323–333.

R1.4 Additionally, I am not sure why genetic drift, and not mutational input, is seen as the causal factor leading to greater mutational robustness. It would seem to be very easy to separate these two—simply perform simulations with a fixed value of NU, decreasing U in concert with increasing N. I think this would be an important test of the authors' interpretations.

Response: We performed the simulations you suggested and confirmed that there is in fact an effect of N independent of mutational supply (Fig 5; lines 244–252).

R1.5 The RNA model, as used here, has the obvious strength of generating epistasis, and I'm certainly not going to suggest any major changes to the core model. But, I'm not sure it's quite the right tool for the job here. The nature of the holey landscape here is quite obscure, and should be sketched out more for the reader. For example, how much potential is there for mutational robustness to evolve given your set-up? Orr's model is based on pairwise interactions–does your model have higher-order epistasis (I would imagine so, to some extent), and how might that affect our results? On the technical side, population sizes are tiny, necessitating high mutation rates. While this is somewhat understandable when using a computationally expensive model, these choices do increase the chances of double-mutations, maybe greatly accelerating the accumulation of DMIs. Certainly, the role of drift is magnified by this small range of population sizes. I feel that the downsides of this model are substantial, if indeed it limits you to these population sizes, and the upsides haven't been fully realized in this paper.

There's a lot to like here, and I think this paper can be a great contribution. I would like to see some response to these points, potentially leading to a little discussion of the limits and characteristics of this complex model.

Response: Taking each point in turn:

(a) "The RNA model, as used here, has the obvious strength of generating epistasis, and I'm certainly not going to suggest any major changes to the core model."

We have extended our earlier work by investigating two additional models: the simpler Russian roulette model introduced by Gavrilets [3,4] (Figs 4 and 5) and a more complex RNA model including intrinsic fitness differences between viable genotypes (Figs 6–8).

(b) "The nature of the holey landscape here is quite obscure, and should be sketched out more for the reader. For example, how much potential is there for mutational robustness to evolve given your set-up?"

We address this question explicitly for the Russian roulette model (line 71). See also response to R3.7.

(c) "Orr's model is based on pairwise interactions—does your model have higher-order epistasis (I would imagine so, to some extent), and how might that affect

our results?"

This question was addressed for the RNA model without intrinsic fitness differences [5]—we found that most IIs are of order higher than pairwise. We added a comparison between the Orr model and our RNA model to the Discussion (lines 560–572).

(d) "On the technical side, population sizes are tiny, necessitating high mutation rates. While this is somewhat understandable when using a computationally expensive model, these choices do increase the chances of double-mutations, maybe greatly accelerating the accumulation of DMIs. Certainly, the role of drift is magnified by this small range of population sizes. I feel that the downsides of this model are substantial, if indeed it limits you to these population sizes, and the upsides haven't been fully realized in this paper."

Some new code and the addition of the Russian roulette model have actually allowed us to run simulations with larger populations (e.g., up to $N = 2000$ in the constant NU regime; Fig 5). However, the realization that divergence—and, therefore, the accumulation of genetic incompatibilities—halt in the large population limit mean that we do not need to run simulations with larger populations (see response to R2.4). For example, populations of $N = 1000$ cannot diverge to the same extent as smaller populations in the fixed U regime (lines 238–240).

On the issue of double mutations, the comparison between the constant NU and constant U simulations shows that the influence of double mutations on our results is negligible. Under $NU = 10$, the runs with $N = 500$ have $U = 0.02$. Thus, a single double mutant is expected to occur every 5 generations in a population. In contrast, in the simulations with $N = 500$ and $U = 0.1$ double mutants occur at a rate 25-fold higher. However, the rates of II accumulation were only sightly different in the two regimes: $b/L = 0.380 \pm 0.015$ with $U = 0.02$ and $b/L = 0.376 \pm 0.012$ with $U = 0.1$ (mean and 95% confidence intervals each based on 200 simulations; Figs 4 and 5).

Notes

R1.6 Lines 11-12: I would turn this around–variation in build-up of RI should correlate with rates of speciation, but there are many other possible contributors.

Response: Done (lines 15–16).

R1.7 Eq. 1–You describe this as "pairs of alleles,", but I think you want to talk about polymorphic loci.

Response: We removed this part of the paper.

R1.8 Lines 55-57: I don't follow why you develop the intro in terms of DMIs, then switch to another focus at the very end of the Introduction? I feel like this paragraph either needs to be moved to Methods, where you can get into the technicalities, or motivated more clearly in the Intro.

Response: We removed the DMI section from the Introduction.

R1.9 Figure 2 is really nice!

Response: Thank you.

R1.10 Lines 108-112: I think you need to say a little bit to explain and justify this fitness function. Intuitively, the nature of selection and the shape/size of the neutral space are clearly quite important to the results. If you're not going to vary its structure or parameters within this paper, we're going to need a really good reason to believe that such variation is not important.

Response: We have addressed this comment in four ways.

(a) We introduced the Russian roulette model and explained how the value of p influences the structure of the neutral network (lines 40–46) and the distribution of values of genetic robustness (lines 70–71).

(b) We changed the RNA folding model to incorporate intrinsic fitness differences between viable genotypes. The simulations summarized in S1 and S2 Figs, when compared to those in Figs 7 and 8, show that intrinsic fitness differences in the RNA model (i.e., the value of σ) have little effect on the results.

(c) We explain that the value of α does not have a large impact on the mean genetic robustness of genotypes in the RNA folding model (lines 134–137).

(d) We show that the parameters we chose for the RNA folding model lead to a realistic distribution of fitness effects (Fig 6; lines 254–268).

R1.11 Lines 206-207: I would call this an "adaptive walk" if the steps are fitness-biased.

Response: We removed that passage. We now explore the Russian roulette model under the Weak Mutation regime (Fig 2; lines 57–63). We explain in detail how the random walk was conducted in the Materials and methods (lines 164–169).

R1.12 Figure 3(a): I think the changes in the intercept here with N should really be addressed in the Results text.

Response: We removed that figure from the paper.

Reviewer #2 (Olivier C. Martin)

R2.1 In computational studies of genotype to phenotype maps, it is common to introduce a fitness for each phenotype because that allows one to tackle (in silico) questions about populations and their evolution on such landscapes. Mutation-selection-drift (and recombination) balance leads to population steady state properties that are quite intuitive and have been mathematically justified in a number of limiting cases. In particular, the smaller the population, the lower the robustness (to mutations). Furthermore, in the presence of recombination, the population also becomes more robust to recombination (thus deleterious epistasis and in particular Dobzhansky–Muller incompatibilities are anti-selected). The present pa-

per takes such a computational framework (based on the genotype to phenotype map when considering predicted RNA secondary structures) to probe the frequency of incompatibilities, motivated by its relevance in allopatric speciation. That motivation led the authors to simulate two independent populations and follow the dependence of the frequency of inter-population incompatibilities on the degree of genotypic "divergence", whereas most works (focusing on robustness, evolvability etc) consider a single population. The authors find that the previously mentioned properties hold as in a single population subject, namely (1) small population sizes lead to lower robustness and (2) recombination leads to a lower rate of deleterious epistasis. [...] From most theorists' point of view, the first two types of results given are exactly as expected.

> **Response:** We believe that these two results are less trivial than you suggest. Taking each of the "properties" in turn:
>
> (1) "In particular, the smaller the population, the lower the robustness (to mutations)."
>
> This result is not quite as well-established as you indicate. Theory and simulations using a quasispecies model [6] and simulations using digital organisms [7] showed that large populations tended to evolve *lower* genetic robustness. We have added a discussion of this point (lines 541–550).
>
> (2) "Furthermore, in the presence of recombination, the population also becomes more robust to recombination (thus deleterious epistasis and in particular Dobzhansky–Muller incompatibilities are anti-selected)."
>
> We agree that this result is well-established for genetic robustness, a point we make in lines 536–541. However, we do not know of any paper making the connection to DMIs explicitly. We made a related point in a paper on a gene network model [8] but in a sufficiently different context (population structure and gene flow) that we don't even cite it in this paper.

R2.2 They also are able to address the dependence of incompatibility with genotypic divergence (neither linear nor quadratic in this model). From most theorists' point of view [this result] is novel as far as I know.

> **Response:** We actually obtained this result previously [5] and therefore decided not to emphasize it in the current paper. But we added a discussion of this point (lines 560–572).

R2.3 My assessment is that the "originality" of the present work relies on the use of two populations.

> **Response:** We propose at least one additional point of originality. The relationship between population size and speciation rate, even in the context of DMIs, is currently unresolved. We make this point more clearly in lines 425–452.

R2.4 I note however that in the large population limit, inter and intra population observables are undistinguishable (a mathematical result that is rather easily shown). Thus this work's originality is confined to the cases of small population sizes.

Response: We incorporated the point about the large population limit (lines 240–243).

R2.5 Overall, the authors have performed a relatively sound computational work but I do object to their fixing the exponent c in Eq 4 by considering the averaging over N: indeed that exponent is different at $N = 1$ and at $N = \infty$ so it makes no sense to do any associated averaging. Unfortunately for the authors, if they accept my objection, the computations of Figs 3 and 4 have to be redone from scratch. Another weakness of their work is that all the simulations are done for RNA molecules of $N = 100$ bases; mathematically, the definition of the snowball exponent c requires one take N to infinity. From the limited simulations presented it is not possible to infer whether the estimates provided for this snowball exponent are meaningful or reliable. The authors may circumvent this difficulty by for instance introducing a measure of snowballing that does not depend on a parametrization such as the one in Eq.4.

Response: We redid that work using new models—see response to R1.5(a). The switch to the Russian roulette model allowed us to reframe that analysis and motivate fitting a linear model instead (starting line 37). We did return to the exponent c in the Discussion but did not fix it (lines 560–572; see response to R2.2).

R2.6 With respect to the issue of originality/novelty of the work, I do see some reasons to be positive but I do not agree with the authors' claim (cf. end of abstract) that they have provided new insights into mechanisms driving speciation.

Response: Noted.

Detailed points

R2.7 The title, abstract and even Fig 1 put forward "holey" landscapes but at the end of the introduction one finds out that it is just a neutral network. I found that way of presenting misleading and recommend they use the term neutral network throughout.

Response: We introduce the concept of a "neutral network" early (line 25 and Fig 1) and make it clear that it is only one particular kind of holey fitness landscape. However, we continue to use "holey fitness landscape" for three reasons. First, because, in our experience, a large proportion of evolutionary geneticists have never heard of neutral networks; but the same is not true for holey fitness landscapes. Second, because we feel that the "neutral" in neutral network has the unfortunate consequence of implying to those unfamiliar with the model that natural selection does not operate. Third, because the new RNA model with intrinsic fitness differences is not a neutral network model, but is still a fitness landscape with "holes".

R2.8 The authors include a section covering "within" vs "between" population effects. Now that they know that in the limit of large population size these two cases are in fact identical (I invite you to include the proof to strengthen the paper), they can streamline this section and improve its scientific content.

Response: See response to R2.4. We have, however, retained the section on IIs within and between populations because we believe it helps understand the small

population regime we focus on.

R2.9 I don't understand why you put your study of incompatibilities in *Drosophila* within the Discussion section rather than have an associated Results subsection.

Response: We moved the *Drosophila* data to the Results (starting line 343).

Reviewer #3

R3.1 This paper argues that large populations with high rates of recombination (high N and high r) will evolve less postzygotic isolation for a given level of genomic divergence. Two distinct reasons are discussed (although they are first clearly distinguished only on lines 292–5):

(A) High-N and high-r populations experience more effective selection for genetic robustness (defined here as tolerance of new mutations). All else being equal, genotypes that are more tolerant of new mutations will also be more tolerant of introgressions at divergent sites. Almost by definition, introgressions into robust genotypes are less likely to cause incompatibilities. (Figs 3–4)

(B) High-r populations are less likely to diverge at sites that are involved in incompatibilities. This is because the incompatibilities may be expressed within populations, slowing the divergence (Fig. 5)

These predictions are compared to comparative evidence from the speciation literature, including two new comparative analyses, which suggest that *Drosophila* clades with higher mean N_e accumulate RI more slowly with genetic distance (Fig. 7), and have more fitness asymmetry in the F1 (Fig. 8).

This is a creative and intriguing paper, but I have some concerns about the presentation and the plausibility of the two mechanisms discussed.

Response: Thank you.

Comments/questions about (A)

R3.2 (A) posits that levels of mutational robustness will vary strongly with N_e and r. I worry that the strength of the effect in the simulations might be an artefact of neglecting variation in fitness among viable genotypes, so that selection for robustness is the only sort of selection that can take place. If other fitness variation were included, I wonder whether populations will evolve genotypes that are intrinsically fitter, regardless of their robustness. Data from *Drosophila* suggest that "speciation genes" involved in DMIs often diverged by positive selection.

Response: We introduced a model with variation in fitness among viable genotypes— see response to R1.5(a). The new data show that variation in fitness does not fundamentally change our results— see response to R1.10(b).

R3.3 Are there within-population data that might test the plausibility of (A)? For example, in the model the lethal mutation rate of a genotype is $U(1-\nu)$, so the model predicts a large

increase in the lethal rate in low recombining regions of the genome, and in low-N_e species. E.g., I think that model predicts that the lethal rate will be much higher in the low-N_e *D. sechellia* than the high-N_e *D. melanogaster*. Are there any relevant data here?

Response: We do not know of data that can be used to test this hypothesis.

Comments/questions about (B)

R3.4 I was unsure how robust prediction (B) would be to variation in the parameters, or the fitness landscape. Previous work by Gavrilets, Fraïsse and others suggests that the presence of potential incompatibilities does not slow down divergence by very much in many cases, and that rates of divergence will depend more on the intrinsic fitness differences between alleles (which are neglected in these simulations).

Response: We repeated the backcross analysis in the RNA folding model with intrinsic fitness differences (Fig 9) and the results were identical to those on the RNA folding model without intrinsic fitness differences (not shown).

R3.5 I would expect the effects of r to also vary with NU. I think that the quantitative treatment, could be much fuller here.

Response: We did not investigate the interaction of the effects of NU and r but we did compare the effect of N under constant U (Fig 4) and under constant NU (Fig 5)—see response to R1.5(d).

R3.6 Rather than the test shown in Figure 5, I think a simpler test and quantification of (B) would be to compare the realized proportion of introgressions that cause incompatibilities, to the expected proportion if sites had diverged at random (which I think is just $(1 - \nu)$).

Response: That test is now conducted implicitly in Figs 4B, 5B, 7B, and 8B where the gray line marks $1 - \nu$. We believe that our more complex test is appropriate because deviations from the expectation of $1 - \nu$ could arise because of a variety of mechanisms.

Wider comments/concerns

R3.7 *The fitness landscape:* The simulations use a fitness landscape based on RNA secondary structure. The properties of this landscape seem important to the results, but are difficult for the reader to understand or compare to previous data and theory (e.g. by Gavrilets). It is not obvious, for example, how many incompatibilities are effectively pairwise (involving 2-sites). Also unclear are the correlations in fitness between neighboring genotypes, the number of paths between fit genotypes etc. I am not sure if these stats are reported in previous work, but in either case, it would be useful to report them here.

Response: On the order of genetic incompatibilities see response to R1.5(c). On the properties of the landscape see response to R1.10.

There is a vast literature on the statistical properties of RNA neutral networks (e.g., correlations [9,10], paths [11,12]). We believe that a review of those statistical properties is beyond the scope of the present paper.

R3.8 My guess is that the computational burden of calculating secondary structure explains why simulations are restricted to very small populations, and to fixed values of certain parameters (e.g. $\alpha = 12$). It is therefore not clear to readers how robust the results are. I wonder whether simpler landscapes with similar properties could be used to extend the results, and provide a fuller exploration of parameter space?

> **Response:** We believe that our results on the Russian roulette model and two versions of the RNA folding model increase the confidence in the robustness of the results. See also the responses to R1.4, R1.5(d), and R1.10.

R3.9 I also wonder why small fitness differences were removed (in eq. 2). If the fitness function is slow to calculate, and prevents an analytical treatment, why use only 0 and 1 fitness values?

> **Response:** We now consider variation in fitness—see response to R1.10(b).

R3.10 *The relevance of the simulations to speciation data:* Simulations use a haploid genotype of 100 sites assumed to represent a single molecule, and a small range of parameters (very small N etc), and often asexuals. Is this appropriate to the empirical work cited, which uses whole genomes of *Drosophila*, *Solanum*, *Mus* etc.?

> **Response:** That is a difficult question and we close the paper with a version of it (lines 573–581).

Minor comments/questions

R3.11 The correlation in Fig. 7 looks impressive, but I wonder whether it is legitimate to ignore variation in N_e between species within each clade. E.g. the *melanogaster* group includes very low N_e species (e.g. *sechellia*) and high-N_e cosmopolitan species (*melanogaster*). It was not clear to me whether, if (A) and (B) are important, the rate of accumulation of RI will vary with the average N_e of the species in the clade.

> **Response:** We added a caveat on this point (lines 482–484).

R3.12 Re. the title, is effect (A) best described as an effect of drift, or does it depend on the population mutation rate, i.e. do results depend on NU or on N independently?

> **Response:** See our response to R1.4.

R3.13 A lot of the past literature cited concerns differences in the rate of evolution of RI that are due to differences in the rate of evolutionary change. But the results here concern differences at a given level of genomic divergence. This important point could be made clearer.

> **Response:** We have calculated the rate of genetic divergence in every regime (see Figs 4C, 5D, 7C, 8D, S1C, and S2D). We have also made this point in the text (lines 229–236).

R3.14 The discussion of the snowball effect is interesting but seems peripheral to the main message of this paper.

> **Response:** We removed the extended discussion of the snowball effect. It is now restricted to lines 560–572.

R3.15 Also, a semantic point, should 1=2?

Response: We do not understand this question.

References

[1] Jablonski D. Species Selection: Theory and Data. Annu Rev Ecol Evol Syst. 2008;39:501–524. doi:10.1146/annurev.ecolsys.39.110707.173510.

[2] Harvey MG, Singhal S, Rabosky DL. Beyond Reproductive Isolation: Demographic Controls on the Speciation Process. Annu Rev Ecol Evol Syst. 2019;50(1):75–95. doi:10.1146/annurev-ecolsys-110218-024701.

[3] Gavrilets S. Evolution and speciation on holey adaptive landscapes. Tr Ecol Evol. 1997;12(8):307–312. doi:10.1016/S0169-5347(97)01098-7.

[4] Gavrilets S. Fitness Landscapes and the Origin of Species. Princeton, N.J.: Princeton Univ. Press; 2004.

[5] Kalirad A, Azevedo RBR. Spiraling Complexity: A Test of the Snowball Effect in a Computational Model of RNA Folding. Genetics. 2017;206(1):377–388. doi:10.1534/genetics.116.196030.

[6] Krakauer DC, Plotkin JB. Redundancy, antiredundancy, and the robustness of genomes. Proc Natl Acad Sci U S A. 2002;99(3):1405–9. doi:10.1073/pnas.032668599.

[7] Elena SF, Wilke CO, Ofria C, Lenski RE. Effects of population size and mutation rate on the evolution of mutational robustness. Evolution. 2007;61(3):666–674. doi:10.1111/j.1558-5646.2007.00064.x.

[8] Whitlock AOB, Azevedo RBR, Burch CL. Population structure promotes the evolution of costly sex in artificial gene networks. Evolution. 2019;73(6):1089–1100. doi:10.1111/evo.13733.

[9] Fontana W, Konings D, Stadler P, Schuster P. Statistics of RNA secondary structures. Biopolymers. 1993;33(9):1389–1404. doi:10.1002/bip.360330909.

[10] Aguirre J, Buldú JM, Stich M, Manrubia SC. Topological Structure of the Space of Phenotypes: The Case of RNA Neutral Networks. PLoS ONE. 2011;6(10):e26324. doi:10.1371/journal.pone.0026324.

[11] Schuster P, Fontana W, Stadler PF, Hofacker IL. From sequences to shapes and back: a case study in RNA secondary structures. Proc R Soc B. 1994;255(1344):279–284. doi:10.1098/rspb.1994.0040.

[12] Reidys C, Stadler PF, Schuster P. Generic properties of combinatory maps: Neutral networks of RNA secondary structures. Bull Math Biol. 1997;59(2):339–397. doi:10.1007/BF02462007.
